# Dataset of working fluid parameters and performance characteristics for the oxy-fuel, supercritical CO_2_ cycle

**DOI:** 10.1016/j.dib.2019.104682

**Published:** 2019-10-19

**Authors:** Andrey Rogalev, Nikolay Rogalev, Vladimir Kindra, Sergey Osipov

**Affiliations:** National Research University “Moscow Power Engineering Institute”, Krasnokazarmennaya Str. 14, Moscow, 111250, Russian Federation

**Keywords:** Supercritical carbon dioxide, Oxy-fuel combustion cycle, Thermodynamic calculation, Air separation unit

## Abstract

This article provides the dataset of working fluid parameters and performance characteristics for the oxy-fuel, supercritical CO_2_ cycle by the thermodynamic optimization procedure employed in the research article “Thermodynamic optimization and equipment development for a high efficient fossil fuel power plant with zero emissions” (Rogalev et al., 2019) [1]. The performance characteristics of the different air separation units, which were used for the investigation of an influence of the produced oxygen purity on power consumption, are presented. Moreover, the methodology for the evaluation of gross and net efficiency is described. The working fluid parameters and performance characteristics are subdivided into several tables differing from each other by the values of initial pressure.

**Table:** Specifications table

Subject area	Engineering
More specific subject area	Thermodynamics
Type of data	Tables, figures
How data was acquired	Numerical simulations based on AspenONE software and FluidProp 9.0 database
Data format	Raw, analyzed
Parameters for data collection	No pretreatment of data was performed. Turbine outlet pressure was fixed at 3 MPa and turbine coolant temperature – at 200 °C during the investigation of the performance characteristics of the oxy-fuel, supercritical CO2 cycle. The pinch-point for the regenerator was equal to 5 °C and the hot air temperature – to 280 °C.
Description of data collection	The simulations have been performed using AspenONE software.
Data source location	Moscow, Russia
Data accessibility	With the article
Related research article	A. Rogalev, E. Grigoriev, V. Kindra, N. Rogalev. Thermodynamic optimization and equipment development for a high efficient fossil fuel power plant with zero emissions. Journal of Cleaner Production https://doi.org/10.1016/j.jclepro.2019.07.067

**Table:** 

**Value of the data** • This data provides working fluid parameters and performance characteristics for the oxy-fuel, supercritical CO_2_ cycle by considering different combinations of the turbine inlet parameters. • The reader can choice among the different turbine inlet parameters combination by evaluating cycle thermodynamic performance. • The reader can choice among the different characteristics of the air separation units to evaluate the performance of the oxy-fuel combustion cycle. • The reader can estimate the difference between gross and net efficiency for the oxy-fuel, supercritical CO_2_ cycle. • The reader can evaluate the influence of the turbine inlet temperature on the coolant mass flow.

## 1 Data

**Fig. 1:**
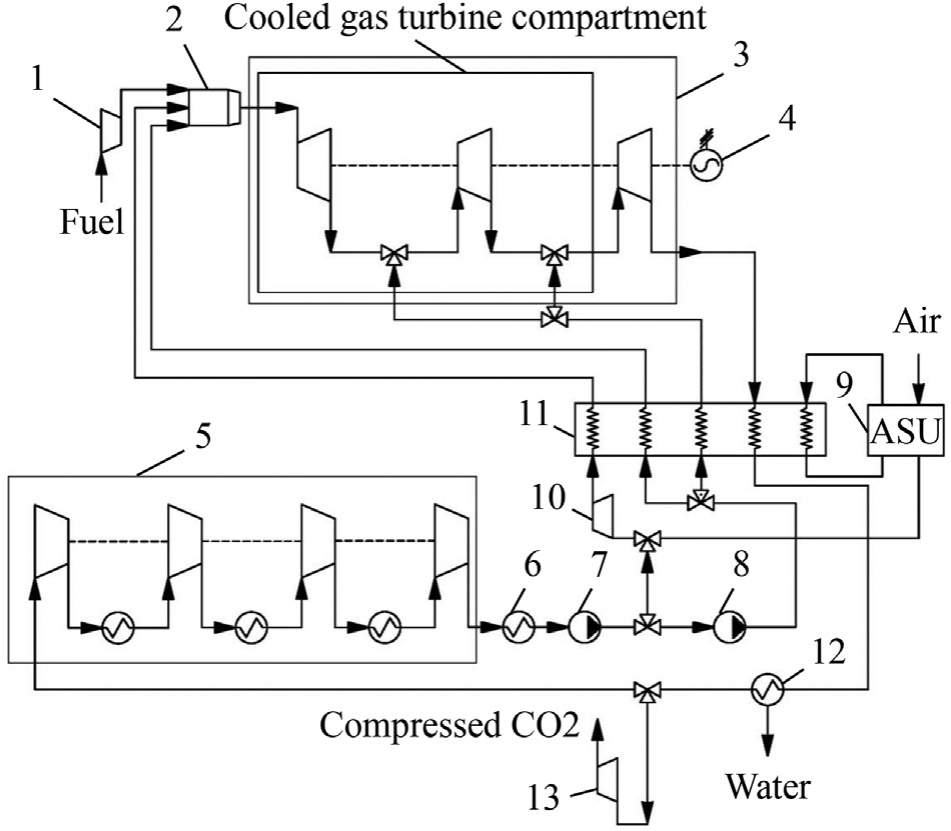
The NET Power cycle.

This article provides the dataset of working fluid parameters and performance characteristics for the oxy-fuel, supercritical CO_2_ cycle, given in Ref. [1]. The concept of the oxy-fuel, supercritical CO_2_ cycle shown in Fig. 1 was proposed in Ref. [2]. The mathematical model of the cooled gas turbine described in Refs. [[3], [4], [5]].

The data for the air separation units (ASU) providing oxygen pressure, temperature, mass flow and purity values, impurities composition and specific power consumption is presented in Table 1, Table 2. The data for the oxy-fuel, supercritical CO_2_ cycle providing for each combination of the turbine inlet pressure and temperature the flow parameters, powers and efficiency levels is presented in Table 3, Table 4, Table 5, Table 6, Table 7.

**Table 1:** The characteristics of high-performance ASU.

№	Oxygen pressure, MPa	Oxygen temperature, °С	Oxygen purity, %	Impurities composition	Specific power consummation of the air separation unit, kWe/(kg O_2_/s)
1	0.12	30	95	3% Ar, 2% N_2_	735
2	–	–	99.5	–	900
3	0.1	–	–	–	900
4	3.5	–	–	–	1512
5	2.7	25	95	3.5% Ar, 1.3% N_2_, 0.1% CO_2_	937
6	2.7	25	90	3.4% Ar, 6.5% N_2_, 0.1% CO_2_	872
7	–	–	95	3% Ar, 2% N_2_	900
8	3.5	–	97	–	1440
9	0.1	–	–	–	972
10	0.24	15	95	3% Ar, 2% N_2_	812
11	4.0	–	–	–	1225
12	2.5	–	85	–	797
	2.5	–	90	–	827
	2.5	–	95	–	878
	2.5	–	97	–	900
	2.5	–	99	–	927
13	3.7	–	–	–	1225
14	–	–	97	–	1109
	–	–	99	–	1157

**Table 2:** Performance characteristics for the industrial ASU.

The ASU model	Air mass flow, kNm^3^/h	Oxygen mass flow, kNm^3^/h	The ratio of the air and oxygen mass flows
KA–5	31.5	5.3	5.94
AK–15p	31.5	5.8	5.43
KA–15	85	15.5	5.48
AKt–30	85	17.5	4.86
KA–32	180	30	6.00
KtA–35	180	34.5	5.22
Kt-70	350	66	5.30
The average ratio of the air and oxygen mass flows			5.46

**Table 3:** The data on thermodynamic analysis of the oxy-fuel, supercritical CO2 cycle for the turbine inlet pressure of 20 MPa.

Natural gas mass flow, kg/s	5,000	5,500	6,000	6,500	7,000	7,500	8,000	8,500
Oxygen mass flow, kg/s	18,61	20,47	22,34	24,20	26,10	27,92	29,78	31,64
Air mass flow, kg/s	102,4	112,6	122,9	133,1	143,6	153,6	163,8	174,0
Turbine coolant mass flow, kg/s	0	0	25,76	75,05	93,7	105,5	127,6	147,9
Relative turbine coolant mass flow, %	0	0	4,100	11,90	14,80	16,60	20,00	23,10
Turbine inlet temperature, °C	669	816	996	1130	1200	1263	1324	1383
Turbine outlet temperature, °C	438	562	688	739	773	799	814	824
Regenerator heated flow outlet temperature, °C	375	501	652	734	768	794	809	819,5
Gross efficiency, %	50,8	57,3	62,2	60,4	58,4	56,2	53,4	50,8
Gross power, MWe	118,2	146,6	173,6	182,6	190,3	196,0	198,8	200,9
Amount of heat realized with a fuel combustion, MWe	232,5	255,8	279,0	302,3	325,5	348,8	372,0	395,3
Net efficiency, %	43,2	49,7	54,6	52,8	50,8	48,5	45,8	43,2

**Table 4:** The data on thermodynamic analysis of the oxy-fuel, supercritical CO2 cycle for the turbine inlet pressure of 25 MPa.

Natural gas mass flow, kg/s	5,500	6,000	6,500	6,750	7,000	7,500	8,000	8,500	9,000
Oxygen mass flow, kg/s	20,50	22,30	24,20	25,13	26,10	27,90	29,80	31,60	33,50
Air mass flow, kg/s	112,6	122,9	133,1	138,2	143,6	153,6	163,8	174,0	184,3
Turbine coolant mass flow, kg/s	0	0	10,50	40,30	74,40	83,20	99,90	118,4	138,0
Relative turbine coolant mass flow, %	0	0	1,660	6,370	11,75	13,10	15,67	18,49	21,48
Turbine inlet temperature, °C	692	808	945	1054	1129	1199	1265	1323	1386
Turbine outlet temperature, °C	433	529	632	689	709	750	776	792	808
Regenerator heated flow outlet temperature, °C	373,5	468	579	663	703,8	744,5	770,5	787,5	803
Gross efficiency, %	50,9	56,4	61,4	63,3	62,2	60,9	58,8	56,4	54,1
Gross power, MWe	130,2	157,4	185,6	198,8	202,4	212,2	218,7	222,8	226,5
Amount of heat realized with a fuel combustion, MWe	255,8	279,0	302,3	313,9	325,5	348,8	372,0	395,3	418,5
Net efficiency, %	43,3	48,8	53,8	55,7	54,5	53,2	51,1	48,7	46,5

**Table 5:** The data on thermodynamic analysis of the oxy-fuel, supercritical CO2 cycle for the turbine inlet pressure of 30 MPa.

Natural gas mass flow, kg/s	5,500	6,000	6,500	7,000	7,250	7,380	7,500	8,000	8,500	9,000	9,500
Oxygen mass flow, kg/s	20,50	22,30	24,20	26,10	26,99	27,46	27,90	29,80	31,60	33,50	35,40
Air mass flow, kg/s	112,6	122,9	133,1	143,6	148,4	151,0	153,6	163,8	174,0	184,3	194,5
Turbine coolant mass flow, kg/s	0	0	0	7,200	28,40	48,60	75,50	82,50	99,90	122,1	138,6
Relative turbine coolant mass flow, %	0	0	0	1,100	4,500	7,700	11,90	12,90	15,60	19,00	21,50
Turbine inlet temperature, °C	600	703	814	934	1019	1083	1134	1208	1268	1330	1394
Turbine outlet temperature, °C	335	422	513	604	651	678	687	731	753	767	787
Regenerator heated flow outlet temperature, °C	285	363	449	544	608	655	682	727	748	762	782
Gross efficiency, %	48,7	53,6	58,1	62,0	63,4	64,1	63,6	63,0	60,7	58,1	56,2
Gross power, MWe	124,6	149,5	175,7	201,7	213,9	219,9	222,0	234,3	239,9	243,3	248,1
Amount of heat realized with a fuel combustion, MWe	255,8	279,0	302,3	325,5	337,1	343,0	348,8	372,0	395,3	418,5	441,8
Net efficiency, %	41,1	46,0	50,5	54,3	55,8	56,5	56,0	55,3	53,1	50,5	48,5

**Table 6:** The data on thermodynamic analysis of the oxy-fuel, supercritical CO2 cycle for the turbine inlet pressure of 35 MPa.

Natural gas mass flow, kg/s	6,500	7,000	7,500	7,750	7,875	8,000	8,500	9,000	9,500
Oxygen mass flow, kg/s	24,20	26,10	27,90	28,90	29,32	29,80	31,60	33,50	35,40
Air mass flow, kg/s	133,1	143,6	153,6	158,7	161,2	163,8	174,0	184,3	194,5
Turbine coolant mass flow, kg/s	0	0	11,70	57,70	61,70	70,90	83,60	109,9	121,1
Relative turbine coolant mass flow, %	0	0	1,840	9,060	9,680	11,11	13,06	17,11	18,78
Turbine inlet temperature, °C	737	840	954	1086	1118	1164	1228	1289	1354
Turbine outlet temperature, °C	434	516	597	652	671	694	724	734	763
Regenerator heated flow outlet temperature, °C	372	450,5	537,5	629,5	654	689	719	728,5	758
Gross efficiency, %	52,9	57,1	60,3	62,6	63,2	63,4	62,0	59,7	57,6
Gross power, MWe	159,9	185,8	210,3	225,8	231,3	235,9	245,2	250,1	254,5
Amount of heat realized with a fuel combustion, MWe	302,3	325,5	348,8	360,4	366,2	372,0	395,3	418,5	441,8
Net efficiency, %	45,3	49,4	52,7	55,0	55,5	55,8	54,4	52,1	50,0

**Table 7:** The data on thermodynamic analysis of the oxy-fuel, supercritical CO2 cycle for the turbine inlet pressure of 40 MPa.

Natural gas mass flow, kg/s	7000	7500	8000	8500	9000	9500	1000	1050
Oxygen mass flow, kg/s	26,10	27,90	29,80	31,60	33,50	35,40	37,20	39,10
Air mass flow, kg/s	143,6	153,6	163,8	174,0	184,3	194,5	204,8	215,0
Turbine coolant mass flow, kg/s	0	0	21,00	57,60	86,70	105,1	129,4	154,0
Relative turbine coolant mass flow, %	0	0	3,300	10,40	13,50	16,30	20,00	23,70
Turbine inlet temperature, °C	779	870	998	1158	1254	1319	1376	1441
Turbine outlet temperature, °C	453	525	607	687	721	743	748	756
Regenerator heated flow outlet temperature, °C	390	458,5	552	667	716	738	743	751
Gross efficiency, %	54,0	57,4	60,7	63,2	62,0	60,3	57,7	55,1
Gross power, MWe	175,9	200,2	225,9	249,8	259,7	266,6	268,3	269,1
Amount of heat realized with a fuel combustion, MWe	325,5	348,8	372,0	395,3	418,5	441,8	465,0	488,3
Net efficiency, %	46,4	49,8	53,1	55,6	54,4	52,7	50,1	47,5

## 2 Experimental design, materials and methods

In the following the configuration of the power plant is firstly described, then the parameters for ASU are given, the method to identify efficiency is described and finally the working fluid parameters and performance characteristics for the oxy-fuel, supercritical CO_2_ cycle are presented.

### 2.1 Power plant configuration

The flow chart of the oxy-fuel, supercritical CO_2_ cycle shown in Fig. 1 is similar to the chart [6]. The gas fuel compressor *1* supplies fuel to the combustor *2* where is also supplied the high purity oxygen produced in the air separation unit *9* and compressed up to the proper pressure by the oxygen compressor *10*. The oxy-fuel mixture combustion increases the flow temperature. The high temperature flow enters turbine *3*, expands and drives the turbine and the electric generator *4*. The exhaust gas flow enters the multi-flow high temperature regenerator *11*, where it transfers its heat to the following three flows:

• oxygen and carbon dioxide mixture traveling to the combustor *2*;
• carbon dioxide recirculation flow upstream the combustor *2* to control the maximal temperature;
• carbon dioxide flow to the turbine *3* cooling.

The regenerator *11* also transfers the low potential heat of the compressed hot air from the air separation unit *9*. After the regenerator *11*, the cooled exhaust gas enters the condenser *12* where the two-component mixture is cooled, water is condensed and removed from the cycle flow. After the condenser *12*, the mixture enriched with carbon dioxide enters the multi-stage intercooled compressor *5*. Upstream the compressor *5* some fluid is taken to the carbon dioxide compressor *13* for its further storage. Downstream compressor *5* the flow enters cooler *6* and then the first stage pump *7*. Apart of the fluid flow is mixed with oxygen on its way to the oxygen compressor *10* and the other part travels to the second stage pump *8*. After the final compression, the flow is split into two parts, one part goes to the combustor and another part is supplied to the turbine cooling. Thus, the cycle is closed.

### 2.2 Dataset for the air separation units modeling

The article [1] shows the dependence of the ASU specific power on oxygen purity obtained by statistical analysis of the characteristics of the different models of air separation units [[7], [8], [9]]. The data on characteristics review of high-performance ASU are presented in Table 1. According to the data, an oxygen purity ranges from 85 to 99.5%, an oxygen pressure differs in a range of 0.1–4 MPa, an oxygen temperature varies in a range of 15–30 °C and the ASU specific power ranges from 735 to 1512 kWe/(kg O_2_/s). The main impurities are argon and nitrogen, the ratio of which varies depending on the ASU scheme and parameters.

The modeling of the oxy-fuel, supercritical CO_2_ cycle presented in Fig. 1 involves evaluation of the amount of low temperature heat released during air compression on the first stage of the oxygen production. To evaluate this value, the performance characteristics of the various industrial ASU were collected in Table 2.

The ratio of the air and oxygen mass flows for the ASU presented in Table 2 is equal to 5.46. This value was used to estimate the amount of low-potential heat supplied in the regenerator.

### 2.3 Net efficiency evaluation method

Unlike traditional gas turbine and combined cycle power plants the oxy-fuel, supercritical CO_2_ cycle is characterized by additional energy penalties on oxygen production and carbon dioxide compression before storage. The value of these penalties in the total losses structure is significant, so the net efficiency is the most suitable parameter to characterize cycle performance. However, gross efficiency was also calculated for the comparison reason. The equations for the energy efficiency coefficients used for calculation of the oxy-fuel, supercritical CO_2_ cycle are presented below.

The cycle gross efficiency is defined as follows (1).

(1) $$\eta_{gross}=\frac{N_{\text{GT}}}{B\cdot Q_{\text{LHV}}},$$

where: *N*_GT_ – the gross power produced by the supercritical carbon dioxide gas turbine, MWe.

*B* – the natural gas mass flow, kg/s;

*Q*_LHW_ – the lower heating value of fuel, MJ/kg.

The cycle net efficiency is defined as follows (2):

(2) $$\eta_{net}=\frac{N_{\text{GT}}-N_{\text{aux}}-N_{\text{ASU}}-N_{CO2}}{B\cdot Q_{\text{LHV}}},$$

where: *N*_aux_ – the electric power for all auxiliary units, excluding ASU and CO_2_ compressor, MWe.

*N*_ASU_ – the air separation unit power consumption, MWe.

*N*_*CO2*_ – the electric power for compression of carbon dioxide before storage, MWe.

The electric power for all auxiliary units *N*_au_ is defined as follows (3):

(3) $$N_{\text{aux}}=N_{MC}+\sum N_{Fuel.C}+\sum N_{Oxy.C},$$

where: *N*_MC_ – electric power for the multi-stage compressor, MWe.

*N*_P_ – electric power for all pumps, MWe.

∑*N*_Fuel.C_ – electric power for the fuel compressor, MWe.

∑*N*_Oxy.C_ – electric power for the oxygen compressors, MWe.

### 2.4 Dataset of working fluid parameters and performance characteristics

The net efficiency data for the oxy-fuel, supercritical CO_2_ cycle presented in Ref. [1] comes from the thermodynamic calculations using AspenONE software. Table 3, Table 4, Table 5, Table 6, Table 7 showed the data on thermal scheme calculation for the different values of the turbine initial parameters.

The data on thermodynamic investigations of the turbine inlet parameters influence upon the oxy-fuel, supercritical CO_2_ cycle net efficiency shows that the maximum value of cycle net efficiency of 56.5% (including air separation unit penalty and carbon capture and storage at 100 bar) is achieved for the turbine inlet temperature of 1083 °C and pressure of 300 bar. The turbine outlet pressure value was fixed at 30 bar and turbine coolant temperature – at 200 °C during the optimization of turbine inlet parameters.

The turbine inlet temperature optimum at a fixed pressure may be explained by specific features of the high temperature regenerator thermodynamic process. Usually, the turbine inlet temperature increase is followed by growth of the cycle mean integral heat intake temperature that increases the equivalent Carnot cycle efficiency, and the cycle thermodynamic efficiency also grows. On the other side, the regenerator analysis shows that the excessive increase of the turbine inlet temperature increases its exhaust temperature that changes the pinch point.

Production and compression of the oxygen supplied to the combustor reduce the cycle net efficiency in average for 7.2%, and the compression of carbon dioxide before storage – for 0.4%. The low energy consumption of CO2 compressor is due to the high cycle minimal pressure, which is equal to 30 bar.

According to the data presented in Table 3, Table 4, Table 5, Table 6, Table 7 the maximal net efficiency of the oxy-fuel, supercritical CO_2_ cycle is achieved for the next combinations of turbine inlet parameters:

• at the 996 °C temperature and 200 bar pressure, the facility net efficiency is 54.6% and the coolant flow coefficient is 3.9%;
• at the 1054 °C temperature and 250 bar pressure, the facility net efficiency is 55.7% and the coolant flow coefficient is 6.6%;
• at the 1083 °C temperature and 300 bar pressure, the facility net efficiency is 56.5% and the coolant flow coefficient is 11.4%;
• at the 1164 °C temperature and 350 bar pressure, the facility net efficiency is 55.8% and the coolant flow coefficient is 11.4%;
• at the 1058 °C temperature and 400 bar pressure, the facility net efficiency is 55.8% and the coolant flow coefficient is 10.5%.
